# The Repurposing Drugs in Oncology (ReDO) Project

**DOI:** 10.3332/ecancer.2014.442

**Published:** 2014-07-10

**Authors:** Pan Pantziarka, Gauthier Bouche, Lydie Meheus, Vidula Sukhatme, Vikas P. Sukhatme, P. Vikas

**Affiliations:** 1 Anticancer Fund, Brussels, 1853 Strombeek-Bever, Belgium; 2 The George Pantziarka TP53 Trust, London KT1 2JP, UK; 3 GlobalCures, Inc, Newton, MA 02459, USA; 4 Beth Israel Deaconess Medical Centre and Harvard Medical School, Boston, MA 02215, USA

**Keywords:** drug repurposing, drug development, metronomics, health policy, health economics, ReDO Project

## Abstract

The Repurposing Drugs in Oncology (ReDO) Project seeks to repurpose well-known and well-characterised non-cancer drugs for new uses in oncology. The rationale for this project is presented, examining current issues in oncological drug development, challenges for health systems, and existing and future patient needs. In addition to discussing the advantages of repurposing, the paper also outlines some of the characteristics used in the selection of drug candidates by this project. Challenges in moving candidate drugs into clinical trial and subsequent practice are also discussed.

## What problem are we trying to solve?

Interest in oncological drug repurposing (also sometimes called drug repositioning) is driven by a range of concerns: productivity issues in current drug development; the need to address existing unmet patient needs; and the economic impact of existing and projected cancer incidence on health systems in both advanced and developing countries. We will briefly touch on each of these, making reference to current trends in oncological drug development in the process, before proceeding to outline the detail of the Repurposing Drugs in Oncology (ReDO) Project.

A recent analysis has shown that the number of new drugs approved per billion US dollars spent on research and development has halved every nine years since 1950, falling around 80-fold in inflation-adjusted terms [1]. Indeed, some observers have described the situation as a ‘productivity crisis,’ [2] and there has been much discussion as to the causes of, and possible solutions to, this crisis [1–4].

The crisis is particularly acute in oncology, where the success rate for new drugs from Phase I trial to US Food and Drug Administration (FDA) approval in the period 2003 to 2011 was around 6.7%, a figure that is about half the rate for non-oncological drugs [5]. The mean development time for antineoplastic drugs, from the time of the first filing of investigational new drug application to the granting of NDA/BLA approval, is estimated to be 8.3 years [6].

This apparent slowdown of new oncology drugs emerging from product pipelines into clinical use is occurring against a twin backdrop, one of increased cancer incidence across the globe, and the other of existing significant therapeutic challenge in many types of malignant disease. The global distribution of cancer incidence is also changing, and this change is projected to continue. Projecting historic demographic trends and changes in cancer incidence, by 2030 cancer incidence in the low to medium human development index (HDI) countries will represent 52% of the global total, or 10.6 million cases [7].This increasing incidence of cancer, associated to a large degree with aging populations in developed countries and with changes in diet, levels of physical activity, and other lifestyle factors in developing countries [8, 9], will increase economic pressures on health systems in both developed and developing economies.

In tandem, and despite successes in some areas, new treatments are needed for refractory disease where there are effective first-line therapies, and effective first-line treatments in some forms of cancer for which current treatment options are limited, such as lung, pancreatic, ovarian, and liver cancers, sarcomas, and other rare malignancies. In particular, there are few effective treatments for the vast majority of metastatic solid tumours, a class of disease that has remained intractable despite notable successes in haematological diseases such as chronic myeloid leukaemia (CML) and some lymphomas [10].

The economic forces arising from increased cancer incidence will exert downward pressure on pricing at a time when costs are rising and the returns on investment are less assured for pharmaceutical companies. Added to this, the fact that the pool of patients able to benefit from new drugs in rare cancers is relatively small compared to the population necessary to produce an adequate return on the investment required to bring a new drug to the clinic, and we have a situation that demands innovative solutions if we are to avoid gridlock.

Before moving the discussion to look at drug repurposing as a strategy to escape that gridlock, mention should also be made of the paradox that the pharmaceutical industry is struggling to successfully develop new oncology drugs at a time when our understanding of cancer at the molecular level is steadily increasing. Paralleling the increasing understanding of cancer at the molecular and genetic level, drug development is increasingly driven by the targeted therapies paradigm. To date, the results from the targeted therapies that have made it into the clinic, for example those targeting the EGFR or VEGF pathways, have been disappointing. Impact on overall survival has often been modest, solid tumours display innate or acquired resistance (even when the tumours are known to be driven by the druggable target of the treatment), side effects can be severe, and costs per patient are high [11, 12].

While some of these problems with targeted treatments may be ameliorated by better stratification of patients, others are inherent in the paradigm itself. A central issue is that tumours are evolutionary adaptive systems, displaying a high degree of intra-tumour genetic heterogeneity [13, 14], and that treatments of all kinds act as selective pressures [15]. Clonal evolution ensures that resistant clones survive and prosper as opposed to those cells which succumb to treatment die-off. In this scenario, therapies which are too closely targeted may well be highly specific and effective as to which clonal populations they attack, but resistance is more likely to evolve as the genetic changes required to escape being a target may be relatively small. For this reason, a number of researchers have suggested that ultimately single-agent targeted therapies will not produce durable responses for the vast majority of tumours [16, 17].

One response to the problems of acquired resistance to targeted agents is to use combination targeted therapies, while another is to use them at an earlier stage of disease, or do both. These approaches are now an area of very active clinical research. In many respects, the hope that was inspired by the initial move to targeted therapies—a hope that has been popularised in the idea of personalised medicine—has transferred to the hope that combinations of targeted agents can be used to precisely attack the specific drivers of cancers in an individual patient. To achieve this future state will require not only a wide range of targeted drugs with low toxicity, and at a cost that makes such combinations feasible, we will also need to be able to identify specific groups of patients that will benefit from these combinations and be able to carry out the necessary clinical trials to prove the efficacy of the treatment protocols. Also, such combinations may involve drugs developed by different companies, with the concomitant logistical and business issues involved in bringing these products together in a single trial. This is no mean feat, and the first-ever trial to investigate a fully personalised approach in metastatic breast cancer has reported disappointing clinical results, even though efficacy was not its primary outcome [18]. Personalised medicine may be feasible but is yet to be proved in practice for the vast majority of tumours, and it is still many years from standard clinical practice.

Of more immediate interest, there has been an increased use of targeted therapy drugs with traditional chemotherapeutic agents [19]. The benefits of this approach in terms of improvements in overall survival are still unclear [11, 20], but it suggests that there is still an important role to play for non-targeted drugs. However, both targeted therapies and traditional chemotherapeutics suffer from toxicity issues which negatively impact patient quality of life, and often require dose reduction, or patient’s withdrawal from treatment. Furthermore, with few notable exceptions, such as imatinib (Gleevec) in CML, the degree of benefit from many approved targeted therapies remains modest, as there have been reports of serious adverse events in some patients [11, 21–24].

While the targeted therapies approach is one that has become dominant in large parts of a pharmaceutical industry, as a society we need to ensure that we do not leave any reasonable opportunity for anti-cancer drug development untapped. There is, therefore, still a need to investigate other sources of anti-cancer agents, including the existing pharmacological armamentarium.

In contrast to the targeted agent paradigm, many classical anti-cancer agents can be considered ‘dirty’ drugs in that they have multiple targets, and in many cases these have yet to be fully elucidated. In the vast majority of cases, the early cancer therapeutics were developed and moved into clinical use before there was a clear indication of the mechanism of action. For example, methotrexate was adopted and used in acute lymphoblastic leukaemia by Sydney Farber and colleagues for more than ten years before Michael Osborn and Frank Huennekens showed that it specifically inhibited dihydrofolate reductase (DHFR) [25]. Indeed, many of these first- and second-generation chemotherapeutics remain the mainstay of treatment in a wide range of cancers and are still being investigated in new combinations (including with targeted agents), in new indications, in new form factors (for example, liposomal formulations), and in new protocols (particularly metronomic).

Drug repurposing is an alternative strategy in drug development, with a history of successful repositioning of existing drugs, largely in non-oncological contexts [26]. The most well-known example is the drug sildenafil (Viagra), originally developed by Pfizer as a treatment for hypertension and angina, which was then repurposed as a successful treatment for erectile dysfunction [27].

This work of extending the use of existing oncology drugs to new cancer indications can be considered a ‘soft’ form of drug repurposing. It aims to short-circuit many of the issues with drug development and testing by taking existing drugs and using them against different indications or in new ways for existing indications. The rationale is clear and is similar to the rationale for the ‘hard’ form of drug repurposing which we are pursuing in the ReDO project. Here, we want to take existing, well-characterised and well-used *non-cancer* drugs and apply them as agents in anti-cancer treatments, either with existing chemotherapeutics or in combination with other repurposed agents.

Drug repurposing, therefore, can be seen as a response to the declining productivity of oncological drug development, as a strategy to reduce development times, and as a source of low-cost treatments to meet the increased demands and unmet needs of cancer patients. It is, in a sense, a very different strategy to the dominant paradigm that guides the development of targeted therapies, but one which may represent a relatively untapped source of novel therapies.

## What are the advantages of drug repurposing?

In contrast to the *de novo* development of new molecules, drug repurposing begins with known pharmaceutical agents with a history of clinical use. There is, therefore, a wealth of data that is accessible to the clinician and researcher, including published data on pharmokinetics, bioavailability, toxicities (common and uncommon), established protocols, and dosing. This is data that is far in excess of what can be derived from Phase I clinical trials of new drugs, particularly for first in class drugs.

This is not to say that we can necessarily avoid Phase I trials of repurposed drugs. Phase I trials may still be required to establish maximum tolerated doses of repurposed drugs if the dosing required to reach oncologically relevant levels is far in excess of the standard doses used in the initial indication of the drug, or if the repurposed drug must be used in untried combinations with other drugs, and for which we must establish that there are no unacceptable toxicities. However, even in the cases when Phase I trials are required, there is a body of existing human data which will inform the structure and dosing schedules of the trial.

Whether trials can proceed directly to Phase II or must be begin at Phase I, it is true to say that drug repurposing is a short-circuit of the extensive drug development process that must take place prior to any Phase I trial. In one review of drug repositioning versus *de novo* drug development, a 10–17 year development lifecycle for *de novo* development is contrasted with a 3–12 year process for repurposed drugs [26]. One of the aims of the ReDO project is to further truncate this development time.

While for pharmaceutical companies the much reduced development lifecycle has direct economic consequences in terms of reduced costs, there are also other potential benefits in terms of risk reduction. As we have seen, the number of candidate drugs that successfully emerge from the product pipeline and into clinical use is below 10% for oncology. It is to be hoped that by reusing existing drugs with known pharmacological properties, the risk of failure can be drastically reduced. However, there are other elements of risk for pharmaceutical companies seeking to repurpose drugs which concern intellectual property rights and patents, and this is a topic that we will return to in the later section of this paper.

Another key advantage of drug repurposing is also related to economics: the use of low cost and/or generic drugs. Given the long development times, high rates of attrition, and low number of successful products delivered, the costs for new oncology drugs, per patient, is very high, typically in the range of $5000–$10,000 per month, or $50,000–$100,000 per course. For example, a recent open letter from over 100 experts in CML decried the costs of three recently approved targeted therapies (bosutinib, ponatinib, and omacetaxine), with costs listed for ponatinib at $138,000 per year, omacetaxine at $28,000 for induction and $14,000 per maintenance course, and bosutinib at about $118,000 per year [28]. This high cost imposes strains on public health systems in advanced economies and is simply unaffordable for the vast number of patients in poor and middle-income countries.

In contrast, many of the drugs being investigated for oncological repurposing are either available as generics or at low cost. Even when these drugs are used in combination protocols with existing standard of care therapies, the incremental costs are likely to be low. While cost alone is not the metric that should decide which therapies are appropriate for patient care, it is an important factor for health systems and insurers, and is a key factor in the development of health policy. In any calculation of cost-utility, interventions with repurposed drugs that have proven efficacy will score more highly than interventions with more costly targeted therapies. It is, therefore, imperative that randomised clinical trials with repurposed drugs are undertaken to prove their efficacy, and hence help reduce the financial burden on stressed health systems, particularly in poorer economies.

This is not to say that there are no potential disadvantages to drug repurposing. It is possible that some drug candidates may show good efficacy, but only at a dosing far in excess of the original registration of the drug, with attendant issues of toxicity, and adverse events (an issue we will return to in the question of clinical trials). More likely the principal disadvantages are related to intellectual property rights and economic incentives, particularly for off-patent and generic drugs—paradoxically also a potential key advantage of repurposing.

While we have focused the discussion on this section largely on economic issues, we should not lose sight of the underlying objective of this project: to identify new interventions that can be brought into clinical use relatively quickly and which can bring about significant clinical improvement to cancer patients. This is particularly the case for patients who have exhausted all existing standard treatments and who have a dismal prognosis. It is this class of patient who is most often a participant in early phase clinical trials, often trials focused on toxicity and dosing, and therefore at high risk of adverse events, and reduced quality of life. It is our contention that such patients should be offered the opportunity to take part in clinical trials of repurposed drugs for which we already have human toxicity data. For these patients participation in a clinical trial that has a lower probability of toxicity and a more manageable risk of adverse events, and subsequent negative impact of quality of life might be a more attractive and humane option.

## A different paradigm

In many respects, cancer has historically been viewed as a disease of the delinquent cell, driven by genomic mutations to express a phenotype characterised by rapid proliferation, altered metabolism, and resistance to normal cell cycle controls. In consequence, much early effort in cancer drug discovery was focused on the development of potent anti-proliferative chemotherapies [10, 25]. Early experiences with leukaemias and lymphomas led to the development of high-dose, multiagent treatment regimens that yielded success with both short-term and long-term remissions. Unfortunately, the experience with solid tumours has not been as positive, perhaps due to the more complex architecture of the solid tumour compared to haematological disease. In the process of gaining understanding for the reason for the relative lack of success with high-dose combination chemotherapies, we have come to learn more about the issues of innate and acquired resistance, genetic heterogeneity, and clonal sub-populations, neo-angiogenesis, tumour hypoxia, immune evasion, metabolism, oncogene addiction, and so on. And yet, much of the focus in oncological drug development remains on the delinquent cell, though the recent excitement around checkpoint inhibitors, a targeted immunotherapy, is a notable exception [29].

Of necessity, high-dose multiagent chemotherapeutic regimens (often described as dose-dense maximum tolerated dose protocols) require breaks in treatment for patients to recover from adverse effects, particularly myelosuppression. While essential for patient survival and quality of life, these interludes also aid in the development of acquired resistance. One response to this has been the development of metronomic chemotherapy, wherein standard chemotherapeutics are used in low doses, and with no (or very short) treatment breaks. Metronomic protocols often use chemotherapy drugs at non-cytotoxic doses, and for extended periods, and the therapeutic effects are thought to be due to an anti-angiogenic and immunomodulatory action [30–34].

In parallel to this development of metronomic chemotherapy as a clinical option, there has also been an increased appreciation of the role of the tumour microenvironment as an integral part of cancer [35]. Rather than cancer being a disease of mutated cells, it is being seen in a more ‘ecological’ light [36, 37], with heterogeneous cell populations within a tumour in close relation to populations of stromal cells of different types: fibroblasts, macrophages, endothelial cells in the tumour vasculature, and so on. An early clinical focus of this improved understanding was the development of anti-angiogenic treatments, both in terms of the development of targeted agents, but also in the understanding that metronomic chemotherapy itself acted anti-angiogenically, and via immune modulation [38–40].

These twin paradigmatic shifts—metronomic dosing and viewing cancer ecologically—will be given priority in our approach to the selection of drugs for repurposing. The intention is not to seek new anti-proliferative cytotoxic agents that can be used at maximum tolerated doses, but to look at drugs from the existing clinical armoury that can be used metronomically or which target the tumour microenvironment or can be used to increase the clinical efficacy of existing cancer therapeutics. In particular, it should be noted that this emphasis on cancer as an adaptive and evolving system also implies that we are not seeking a ‘magic bullet’ or a targeted agent. Rather, there is a clear rationale for seeking combinations of agents—both repurposed and standard oncological drugs—which together work to attack multiple aspects of the tumour, and the microenvironment.

In the simplest cases we are seeking to repurpose drugs to add to existing regimens and protocols because they improve clinical efficacy, for example by reversing drug resistance, or altering an aspect of the microenvironment such that chemotherapeutic drug bioavailability is improved or toxicity reduced. However, we are also proposing that combinations of repurposed drugs might also have significant therapeutic effect in and of themselves, and that these combinations may be as, or even more, effective as some standard of care therapies, but with lower toxicity and at lower cost.

## What will ReDO do?

High throughput drug screening, *in silico* modelling, and other techniques are identifying an ever increasing number of compounds—both novel and existing—with some level of anti-cancer activity. There is no shortage of drug candidates for repurposing, in fact the opposite problem exists, and there are too many candidate drugs with *some* potential activity that warrants *some* degree of investigation. However, there are candidate drugs for which there is often a higher degree of evidence—*in vitro* and/or *in vivo* studies, *in silico* modelling, isolated case reports, or early phase clinical trials. Often, the evidence is dispersed, unsummarised, or otherwise obscured.

If we are to make progress with drug repurposing and bring these drugs to the clinic, then a number of steps have to be taken:

1. Candidate drugs have to be filtered and assessed and the most promising identified for further clinical investigation.
2. The data for these most promising drugs need to be reviewed, summarised, and brought to the attention of clinical investigators and the broader oncological community.
3. Identification and documentation on how these drugs can be combined with existing therapies, or with other repurposed drugs, for specific cancer indications must be developed.
4. Work must take place with investigators to develop clinical trials to provide positive or negative evidence of efficacy.
5. Where necessary, we must suggest areas where further pre-clinical work is necessary to shed further light on mechanisms of action, evidence of synergy for combinations, or seek other additional data to help prioritise those agents, or combinations with a high probability of clinical efficacy.

The ReDO project aims to undertake these steps and to work with clinicians to make these trials a reality. A key output from this project, therefore, will be a series of papers that focus on specific drug candidates, and which will make the case for specific clinical trials by reviewing and summarising existing data, and formulating sample combinations and protocols for the treatment of various cancer types.

Much current work on drug repurposing in oncology focuses on single agents or on specific cancer types. In contrast, ReDO will focus on a wide range of agents and across many different cancer indications, examining evidence not just for efficacy as single agents, but seeking evidence for drug combinations in those indications. These combinations may include multiple repurposed drugs working in concert, as well as existing therapeutics. In addition, the scope of the project extends beyond the purely clinical, and encompasses the wider social and political issues that impact the success, or otherwise, of repurposing.

## Candidate drug characteristics

The drugs that we have selected as most promising share a number of common characteristics, namely:

- They are well-known drugs, with many years of widespread clinical use, rather than newer agents recently brought into clinical use for non-cancer indications. Often, they are available as generics, but this is not a primary consideration.
- The toxicology profile is good, with low toxicity even with chronic dosing. Use for metronomic protocols is seen as an advantage, though no drug is ruled out if it cannot be used in such a schedule.
- There is a plausible mechanism of action. Note that a drug need not be directly cytotoxic, candidate drugs may have putative mechanisms of action that are anti-angiogenic, inhibit particular pathways, or target aspects of the tumour microenvironment.
- Strong evidence: *in vitro*, *in vivo*. and human data (epidemiological, published case reports, clinical trials). Human data is scored significantly more highly than *in vivo* or *in vitro* work; results in syngeneic, orthotopic mouse models have the highest weight in pre-clinical work.
- There is evidence of efficacy at physiological dosing. There are many drugs where there is pre-clinical work that shows efficacy but at doses, or by route of administration, not achievable in patients, or only achievable at doses with significant toxicity.

The first six drugs to be investigated by the ReDO project are listed in Table 1. They were selected based on the above criteria from a list of more than sixty non-oncological drug candidates with recent evidence of anti-cancer activity. Other candidate drugs on our list include: EPA/DHA, PUFAs, losartan/ARBs, chloroquine/hydroxychloroquine, statins, propranolol/beta blockers, omeprazole/PPI, and polysaccharide K (PSK).

It may be noted that the list does not include a number of non-cancer drugs which have increasingly attracted attention from the oncological community, for example, the anti-diabetic drug metformin or aspirin. Metformin has become a significant object of clinical cancer research in recent years, driven in part because of the strong epidemiological evidence that it reduces cancer risk in patients with type II diabetes [41], which has also spurred on significant pre-clinical and clinical exploration [42–44]. This level of interest has moved from the laboratory to the bedside, and there are now more than fifty trials investigating the use of metformin in cancer treatment, as well as prevention. It is clear then, that the repurposing of metformin as an anti-cancer agent has moved into the mainstream, and has no need of further effort to make the case for it.

Aspirin is also missing from our list despite significant levels of interest in the anti-cancer properties that it may possess. While there is some evidence that aspirin may have some influence on cancer treatment post-diagnosis [45, 46], the bulk of attention has been on the prophylactic use of aspirin, including for those at high risk due to Lynch Syndrome [47], or from previous incidence of colorectal cancer (CRC) [48]. And, as with metformin, there is also a wide range of pre-clinical and clinical activity around aspirin and cancer such that there is little that a project such as ours could do to add to the debate, in contrast to the situation with lesser investigated drugs such as mebendazole, nitroglycerin, or cimetidine.

## What’s in the way of starting trials?

While the focus of the ReDO project is on providing clinicians with translationally relevant data, we are also aware that there are multiple human, institutional, and economic factors at work. In some respects capturing the interest and the imagination of clinicians is the easier part of this project. More intractable are the institutional and economic factors.

The majority of clinical trials, across all areas of medicine, are sponsored by pharmaceutical companies seeking a return on the investments they have made in developing the drug being trialled. In the case of repurposed drugs, which are often generic or else towards the end of patent life-time, the financial incentives to go to trial do not exist. Yet without the evidence from clinical trials, particularly randomised controlled trials, the potential economic and medical benefits that will accrue from the adoption of these low-cost repurposed drugs will not be realised. Society as a whole loses from this impasse.

In the short-term there are a number of things that we can do to ameliorate the situation. For example smaller clinical trials are easier to organise, less expensive to administer, and can proceed more quickly. The downside is lower patient numbers, lower levels of statistical power, and problems with patient accrual (particularly acute for rarer cancers). However, these smaller trials can add to the weight of evidence in favour of repurposed drugs (assuming positive results). There is also scope for the not-for-profit sector, particularly for government-funded bodies and health systems, to fund these clinical trials in the absence of pharmaceutical company involvement.

One of the organisations involved in the ReDO project, the Belgian not-for-profit organisation the Anticancer Fund, is already involved in funding a number of such trials. Examples include the Ketorolac in Breast Cancer Surgery trial (NCT01806259), a trial of Nitroglycerin in non-small cell lung cancer (NCT01210378) and the Fluvabrex trial (NCT02115074), investigating the combination of fluvastatin and celecoxib in children with refractory optic-pathway glioma. However, there is considerable scope for many more such trials to take place, and given the potential economic benefits to stressed health systems, it is in the interests of these systems to make the investments in proving the efficacy of low-cost therapeutics. There are, of course, also indirect social and economic benefits in that treatments with a lower burden of morbidity incur fewer costs to society, as well as offering improved quality of life to patients and their families.

In the longer term there is perhaps a need to develop new models of intellectual property rights and/or incentivisation schemes such that these drugs can be piloted through the trials process with the involvement of pharmaceutical companies, health insurers, not-for-profit organisations, and patient-driven lobby groups.

A recent blog for the journal Health Affairs [49] made the case that in the same way that society has recognised the incentivisation issue at the heart of drug development for orphan diseases—with the widespread adoption of ‘orphan drug’ legislation across the world – so too there is an incentive issue with regards to repurposing existing drugs as oncological therapies. Financial orphans include drugs or therapies for which there is little or no patent protection, in other words the bulk of the drugs we view as candidates for repurposing in oncology. There is a case, therefore, for innovation, including changes in legislation, to address the lack of incentives such that society can reap the benefits of repurposing.

## Phase II, phase III, or something else?

There is a trend towards ever larger Phase III trials in oncology, a trend driven by the slight incremental improvements in outcome measures (overall survival or progression free survival) from some new drugs. These small response improvements necessitate larger sample sizes to achieve sufficient power [22, 50]. From the perspective of drug repurposing this trend is problematic due to the logistics and the costs involved in developing and running a large Phase III trial. Without significant sponsorship, the costs in time, and personnel to develop and run a large, well-powered, multi-centre randomised controlled trial are likely to be beyond the resources available to the often small groups of clinicians involved in drug repurposing research.

From a purely practical point of view, therefore, smaller trials are more likely for repurposed drugs and these are often designated as Phase II trials, which has lower status in the eyes of many clinicians, even though the trials may be of a high quality, be properly randomised, and have positive results on clinically relevant endpoints. Rather than thinking in terms of Phase III trials, perhaps we need to think in terms of Phase II/III trials, or ‘pivotal’ clinical trials in the case of repurposing. In these cases, the results from randomised controlled trials, with smaller patient numbers than is common in many current Phase III trials, should be accorded a similar level of recognition by the oncological community to that afforded to large industry-funded trials.

This has been recognised to some extent by the FDA in the United States with the new ‘breakthrough therapy’ designation, a move explicitly designed to expedite the approval, and clinical adoption of new therapies for serious or life-threatening conditions [51]. To date similar legislative recognition of the need to accelerate drug approval for such conditions has not been afforded in the European Union.

The concern about the reliance on large Phase III trials is not simply a theoretical one, there have been a number of cases where clinical trials have reported positive results with repurposed drugs, and yet the results have not lead to any change in clinical practice. For example, there have been numerous clinical trials involving the use of the histamine type 2 receptor antagonist, cimetidine in CRC [52], with a recent Cochrane review concluding that ‘cimetidine appears to confer a survival benefit when given as an adjunct to curative surgical resection of colorectal cancers.’ [53] Yet, the results from these various trials have not been translated into clinical practice with the consequence that the comparison treatments cimetidine has been compared with no longer represent standard of care. Similarly, a recent trial of the antifungal agent itraconazole in NSCLC had to be terminated early, despite positive results, because the standard of care had changed during accrual [54]. In both cases it is unlikely that industry-funded trials would have suffered the same fate.

In addition to funding, pharmaceutical companies provide for a pool of advocates for the drugs they develop, in partnership with clinicians and academics, they ensure that positive results build momentum, and they guide the process towards successful trial completion, drug licensing, and eventual incorporation into clinical practice. For repurposed drugs this pool of advocates is largely missing.

For instance, data from a trial performed in NSCLC with nitroglycerin [55] and in CRC with cimetidine [53] seem to offer sufficient evidence for a large effect in serious diseases. However, without the impetus and the funding from a sponsor—normally a pharmaceutical company—there is no one pushing for official approval, market authorisation, or the inclusion in official guidelines. Even when the evidence reaches the highest possible level, this absence of official approval means results are ignored, to the detriment of cancer patients, and public health in general. What could be called “practice-changing” trial results actually do not change practices on their own.

Aspirin is the prototypical example of such a problem. In 2003, Sandler *et al* reported the early termination of a randomised placebo-controlled trial of aspirin after the efficacy boundary was crossed at interim analysis [48]. In this trial, patients curatively treated for CRC had substantially fewer new colorectal adenomas if they were in the aspirin arm. Additional positive evidence has been gathered since this report [56], but aspirin is not mentioned in the 2014 National Comprehensive Cancer Network (NCCN) guidelines and is briefly discussed in the 2013 European Society for Medical Oncology (ESMO) guidelines, but actually in relation to the less clinically-relevant evidence from recent molecular findings in the adjuvant setting [57].

## Off-label usage?

Finally, we acknowledge also that the data we make available as part of the ReDO project will provide clinicians treating late stage cancer patients with additional avenues to explore in an off-label, off-trial setting. The practice of off-label usage of drugs in oncology varies according to legal framework, institutional setting, and cultural norms. In the UK, the Medical Innovation Bill, should it become law, may have an impact on the prevalence of this practice. Other factors which have an influence on the degree of off-label drug usage in oncology include the use of medical decision support systems, and the attendant procedures entailed in stepping outside of programmed boundaries, particularly in the context of a capitated reimbursement system.

In addition to potentially providing direct patient benefit, such off-label usage of repurposed drugs provides important and clinically relevant data in the form of well-documented case reports, some of which make up the evidence we draw upon in the ReDO project. Indeed, clinicians have played a major role in identifying and investigating repurposed drugs, but the concept of ‘field discovery’ is one that is generally underestimated [58].

Additionally, there is scope to do more with such experiences through the collection and sharing of data. For example, the US not-for-profit organisation GlobalCures, which is part of the ReDO project, has initiated a programme, called SHARE, explicitly to collect such clinical information.

## Conclusion

This paper has outlined a number of issues with current oncological drug development, economic pressures imposed on health systems due to increased costs of new cancer treatments, and the increasing incidence of cancer, both in developed and developing countries. Drug repurposing of existing non-oncological agents, particularly low-cost and generic drugs with known toxicity profiles has been proposed as a strategy to address these issues. While the advantages of repurposing are many, and have been outlined above, we should focus in this concluding remark on the ultimate benefit that we are seeking.

The authors of this paper are a diverse group of researchers, clinicians, and patient advocates all working in the not-for-profit sector. We seek new treatments that meet the needs of existing patients in as short a time-frame as possible and at a cost that is affordable both in developed and developing countries. Most of all we seek treatments that are at least as efficacious as existing standard of care treatments, including the newer targeted therapies which are emerging into clinical practice, but with lower toxicity and offering an improved quality of life to patients. There are numerous hurdles to overcome to make drug repurposing a reality, but perhaps the first of these is in convincing clinicians and patients alike that there really are old drugs already in the pharmacist’s cabinet which can provide some value to cancer patients in fighting their disease. We hope that this paper, and those that accompany it and focus on individual drugs, can provide the scientific rationale and the evidence that this is the case.

## Author contributions

Primary author: Pan Pantziarka. Contributing authors: Gauthier Bouche, Lydie Meheus, Vidula Sukhatme, and Vikas P. Sukhatme. All authors read and approved the final manuscript.

## Competing interests

All the authors are associated with not-for-profit organisations that aim to repurpose drugs for oncology treatments. Vikas P. Sukhatme is also on the Scientific Advisory Boards and a consultant to Berg Pharma and Mitra Biotech. The other authors have no competing interests to declare.

## Figures and Tables

**Table 1.** The first six drugs to be assessed by the ReDO project

Drug	Type	Existing Indication	Availability
Mebendazole	Anthelminthic	Threadworm infections	Generic
Nitroglycerin	Vasodilator	Angina	Generic
Cimetidine	H2-receptor antagonist	Peptic ulcer	Generic
Clarithromycin	Antibiotic	Respiratory tract infection	Generic
Diclofenac	NSAID	Pain relief	Generic
Itraconazole	Antifungal	Broad spectrum antifungal	Generic
